# Symptomatic and asymptomatic candidiasis in a pediatric intensive care unit

**DOI:** 10.1186/1824-7288-37-56

**Published:** 2011-11-21

**Authors:** Ali Ertug Arslankoylu, Necdet Kuyucu, Berna Seker Yilmaz, Semra Erdogan

**Affiliations:** 1 Department of Pediatric Intensive Care, Mersin University School of Medicine, Mersin, Turkey; 2 Department of Pediatric Infectious Diseases, Mersin University School of Medicine, Mersin, Turkey; 3 Department of Pediatrics, Mersin University School of Medicine, Mersin, Turkey; 4 Department of Biostatistics, Mersin University School of Medicine, Mersin, Turkey

**Keywords:** Candida, candidiasis, pediatric intensive care unit

## Abstract

**Introduction:**

This study aimed to examine the incidence, epidemiology, and clinical characteristics of symptomatic and asymptomatic candidiasis in a pediatric intensive care unit (PICU), and to determine the risk factors associated with symptomatic candidiasis.

**Methods:**

This retrospective study included 67 patients from a 7-bed PICU in a tertiary care hospital that had Candida-positive cultures between April 2007 and July 2009. Demographic and clinical characteristics of the patients, Candida isolates, antimicrobial and antifungal treatments, and previously identified risk factors for symptomatic candidiasis were recorded, and symptomatic and asymptomatic patients were compared.

**Results:**

In all, 36 (53.7%) of the patients with Candida-positive cultures had asymptomatic candidiasis and 31 (46.3%) had symptomatic candidiasis. Candida albicans was the most common Candida sp. in the asymptomatic patients (n = 20, 55.6%), versus Candida parapsilosis in the symptomatic patients (n = 15, 48.4%). The incidence of central venous catheter indwelling, blood transfusion, parenteral nutrition, and surgery was higher in the symptomatic patient group than in the asymptomatic patient group (P < 0.5). Surgery was the only independent predictor of symptomatic candidiasis according to forward stepwise multivariate logistic regression analysis (OR: 6.1; 95% CI: 1.798-20.692).

**Conclusion:**

Surgery was the only risk factor significantly associated with symptomatic candidiasis and non-albicans Candida species were more common among the patients with symptomatic candidiasis. While treating symptomatic candidiasis in any PICU an increase in the incidence of non-albicans candidiasis should be considered.

## Introduction

Candida infections are among the major nosocomial infections associated with excessive morbidity and mortality, prolongation of hospital stay, and increased healthcare costs [1]. As the length of stay in intensive care units (ICUs) and the frequency of invasive procedures increase, the incidence of Candida colonization and Candida infection increases. Due to the severity of their primary disease and suppressed immune system patients in ICUs are at high risk of Candida infections because of invasive monitoring techniques and the specific therapeutic procedures used. Most patients suffer no ill effects due to Candida colonization because of its low-level virulence; however, in some patients with suppressed defenses the organisms invade and cause illness [2].

One study that included 20 pediatric intensive care units (PICUs) in 8 countries reported that fungi were the third most frequent infection agents, following bacteria (68%) and viruses (22%) [3]. Among fungi, Candida spp. are the most frequent causes of fungal infections in PICUs [4]. Few data exist on candidiasis in PICUs in developing countries. Most of the literature on candidiasis primarily concerns adult ICU patients, and differences in epidemiology, Candida spp., and the distribution, management, and outcome of candidiasis between adults and children limits extrapolation of the conclusions to children. The present study, therefore, aimed to determine the incidence, epidemiology, and clinical characteristics of symptomatic and asymptomatic candidiasis in a PICU, and to determine the risk factors associated with symptomatic candidiasis.

## Materials and methods

This retrospective study, which was carried out in a 7-bed PICU of a tertiary care hospital, included the patients who had positive cultures for Candida spp between April 2007 and July 2009. The yearly number of admissions to the PICU was 248 during the study period. In the PICU the protocols recommended by Centers for Disease Control and Prevention (CDC) are applied to prevent sepsis. Patient data were obtained from PICU records and the hospital infection committee. Approval of the study was obtained by the local Institutional Review Board with waiver of informed consent since the sudy did not involve therapeutic interventions or potential risks to involved subjects.

Cultures were sent if infection was suspected at any time after admission to PICU. In addition, surveillance cultures were sent for all patients after 5 days of stay in the PICU if the patient had any of the previously known high-risk factors for candidiasis such as broad-spectrum antibiotics, endotracheal intubation, mechanical ventilation, intravenous catheters, presence of multiple-organ failure, and corticosteroid therapy. Patient demographics, clinical presentation and primary diagnosis, presence of an indwelling catheter, broad-spectrum antibiotic use, endotracheal intubation, corticosteroid therapy, and blood transfusion were recorded. The site from which culture samples were obtained, duration of hospitalization before the culture sample was obtained, and whether or not patients were symptomatic at the time samples were obtained were noted. Surgical procedures, length of stay in the PICU, and the mortality rate were also recorded. Patients were categorized as symptomatic and asymptomatic. Symptomatic candidiasis was defined as candidiasis with symptoms and signs of sepsis (fever > = 38°C, tachypnea, tachycardia, and leukocytosis), respiratory distress, or shock, either at the time sample was taken for fungal culture or anytime within 72 hrs of it. Asymptomatic patients of candisiasis were those who had no symptom or sign of sepsis but had cultures positive for Candida. The clinical characteristics of the symptomatic and asymptomatic patients were investigated.

### Statistical analysis

SPSS v.11.5 was used for statistical analysis. The Shapiro-Wilk test was used to determine if the continuous variables were normally distributed. The Mann-Whitney U test was used for continuous variables, and Spearman's chi-square test or the likelihood ratio test was used for categorical variables. Descriptive statistics (median and 25th-75th quartiles) for continuous variables, and the number and percentages for categorical variables are given.

## Results

The study included 67 patients (41 males, 26 females) with Candida-positive cultures which were obtained from all sites shown in Table 1. There weren't any differences between the symptomatic and asymptomatic patient groups in terms of age or gender. In all, 36 (53.7%) of the patients with Candida-positive cultures had asymptomatic candidiasis and 31 (46.3%) had symptomatic candidiasis. In total, 36 of the patients did not receive antifungal treatment. The antifungal agents used to treatment the patients with symptomatic candidiasis were fluconazole (n = 12, 17.9%), caspofungin (n = 8, 11.9%), amphotericin-B (n = 6, 9.0%), and voriconazole (n = 4, 6%). In all, 38 of the patients (56.7%) survived and 29 (43.3%) died. During the study period the mortality rate of whole PICU was 14%. Antibiotic treatment (ampicillin sulbactam 29.9%, ceftriaxone 22.4%, sulperazone 19.4%, meropenem 7.5%, ciprofloxacin 6%, vancomycin 2%, amikacin 2%, piperacillin tazobactam 2%, clarithromycin 2%) was administered to 65 (97%) of the patients.

**Table 1:** Descriptive statistics and demographic characteristics of the patients with Candida-positive cultures.

		n	%
Age (months)	0-3	2	3.0
	
	4-12	29	43.3
	
	13-60	21	31.3
	
	60 +	15	22.4

Culturing site	Blood	26	38.8
	
	Urine	19	28.4
	
	Feces	4	6.0
	
	Catheter	9	13.4
	
	Wound	1	1.5
	
	CSF	1	1.5
	
	Pharynx	2	3.0
	
	Other	5	7.5

Candida spp.	Albicans	28	41.8
	
	Tropicalis	5	7.5
	
	Parapsilosis	23	34.3
	
	Glabrata	4	6.0
	
	Kefyr	2	3.0
	
	Sake	2	3.0
	
	Kefyr + albicans	1	1.5
	
	Albicans + tropicalis	2	3.0

Central venous catheter	No	18	26.9
	
	Femoral	45	67.2
	
	Jugular	2	3.0
	
	Femoral + Jugular	2	3.0

Urinary catheterization	No	5	7.5
	
	Yes	62	92.5

Mechanical ventilation	No	15	22.4
	
	Yes	52	77.6

Parenteral nutrition	No	21	31.3
	
	Yes	46	68.7

Blood transfusion	No	8	11.9
	
	Yes	59	88.1

Corticosteroid use	No	39	58.2
	
	Yes	28	41.8

Surgery	No	47	70.1
	
	Yes	20	29.9

Broad-spectrum antibiotic use	No	2	3.0
	
	Yes	65	97.0

The patient's descriptive statistics and demographic characteristics are given in Table 1. Mean age of the patients was 36.3 ± 42.4 months. Mean duration of broad-spectrum antibiotic use was 28.6 ± 37.2 days, mean length of stay in the PICU was 50.9 ± 68.0 days, and mean length of time between admission and Candida growth in culture was 19.8 ± 23.4 days. The asymptomatic and symptomatic candidiasis groups were compared in terms of demographic characteristics; age, central venous catheter indwelling, blood transfusion, parenteral nutrition, and surgery statistically differed. A comparison of the asymptomatic and symptomatic candidiasis groups in terms of demographic characteristics and P values is shown in Table 2.

**Table 2:** Comparison of the asymptomatic candidiasis and symptomatic candidiasis groups, in terms of demographic characteristics and P values.

		Asymptomatic Candidiasis		Symptomatic Candidiasis		P
	
		Number	%	Number	%	
Age (months)	0-3	0	0.0	2	6.5	0.014
		
	4-12	17	47.2	12	38.7	
		
	13-60	15	41.7	6	19.4	
		
	60+	4	11.1	11	35.5	

Gender	Female	13	36.1	13	41.9	0.626
		
	Male	23	63.9	18	58.1	

Candida spp.	Albicans	20	55.6	8	25.8	0.204
		
	Tropicalis	2	5.6	3	9.7	
		
	Parapsilosis	8	22.2	15	48.4	
		
	Glabrata	3	8.3	1	3.2	
		
	Kefyr	1	2.8	1	3.2	
		
	Sake	1	2.8	1	3.2	
		
	Kefyr + Albicans	0	0.0	1	3.2	
		
	Albicans + Tropicalis	1	2.8	1	3.2	

Central venous catheter	No	17	47.2	1	3.2	< 0.0001
		
	Femoral	18	50.0	27	87.1	
		
	Jugular	1	2.8	1	3.2	
		
	Femoral + Jugular	0	0.0	2	6.5	

Broad-spectrum antibiotic use	No	2	5.6	0	0.0	0.111
		
	Yes	34	0.0	31	100.0	

Blood transfusion	No	7	19.4	1	3.2	0.060
		
	Yes	29	80.6	30	96.8	

Corticosteroid use	No	19	52.8	20	64.5	0.331
		
	Yes	17	47.2	11	35.5	

Parenteral nutrition	No	21	58.3	0	0.0	< 0.0001
		
	Yes	15	41.7	31	100.0	

Surgery	No	30	83.3	17	54.8	0.011
		
	Yes	6	16.7	14	45.2	

Prognosis	Survived	22	61.1	16	51.6	0.434
		
	Died	14	38.9	15	48.4	

The percentage of patients with an indwelling catheter was significantly higher in the symptomatic group than in the asymptomatic group (P < 0.0001). There was no difference in the number of patients with femoral and jugular central venous catheters between the groups. Broad-spectrum antibiotic use was not statistically different between the groups (P = 0.111), whereas parenteral nutrition was (P < 0.0001). Although 58.3% of the patients in the asymptomatic group were not administered parenteral nutrition, all the patients in the symptomatic candidiasis group received parenteral nutrition. The difference between the 2 groups in terms of surgery was statistically significant (P = 0.011), whereas the Candida spp. indentified in the 2 groups did not statistically differ (Table 2). When we compared the distribution of candida species in blood stream vs non-blood stream isolates, non-candida albicans blood stream infection was significantly high (p = 0.001). And the common site of isolation of candida parapsilosis was blood. Pediatric risk of mortality score (PRISM) of the patients with symptomatic candidiasis was 44.61 ± 21.09 and this value was 30.42 ± 17.05 for the patients with asymptomatic candidiasis. There was a significant difference between groups (p = 0.003).

When the patients were divided into 2 groups according to prognosis their demographic characteristics were compared and there was a statistically significant difference between the patients that died and survived, in terms of central venous catheters, blood transfusion, and parenteral nutrition. The descriptive statistics for these results are given in Table 3. The duration of stay in the PICU before Candida growth in culture, total mechanical ventilation period, period of broad-spectrum antibiotic use, and duration of hospitalization in the PICU were significantly longer in the symptomatic candidiasis group than in the asymptomatic group. The descriptive statistics for these parameters are given in Table 4. There was no significant difference between the ventilated and non ventilated patients in terms of site of the culture (p = 0.092).

**Table 3:** Comparison of the patients that survived with the patients that died, in terms of demographic characteristics and P values.

		Survived		Died		p
	
		Number	%)	Number	Percentage (%)	
Age (Months)	0-3	0	0.0	2	6.9	0.209
		
	4-12	18	47.4	11	37.9	
		
	13-60	13	34.2	8	27.6	
		
	60 +	7	18.4	8	27.6	

Gender	Female	11	28.9	15	51.7	0.058
		
	Male	27	71.1	14	48.3	

Central venous catheter	No	14	36.8	4	13.8	0.017
		
	Femoral	22	57.9	23	79.3	
		
	Jugular	0	0.0	2	6.9	
		
	Femoral + Jugular	2	5.3	0	0.0	

Broad-spectrum antibiotic use	No	2	5.3	0	0.0	0.502
		
	Yes	36	94.7	29	100.0	

Blood transfusion	No	8	21.1	0	0.0	0.008
		
	Yes	30	78.9	29	100.0	

Corticosteroid use	No	24	63.2	15	51.7	0.347
		
	Yes	14	36.8	14	48.3	

Parenteral nutrition	No	16	42.1	5	17.2	0.030
		
	Yes	22	57.9	24	82.8	

Surgery	No	28	73.7	19	65.5	0.469
		
	Yes	10	26.3	10	34.5	

**Table 4:** Descriptive statistics of measurements and their P values for the symptomatic and asymptomatic groups.

	Asymptomatic Candidiasis			Symptomatic Candidiasis			P
	
	**Min-Max**.	Mean rank	Median (25%-75%)	**Min-Max**.	Mean rank	Median 25%-75%)	
Duration of stay in PICU before culturing time	1-59	24.6	9 (2-16.5)	1-132	44.92	23 (15-37)	< 0.0001

Duration of mechanical ventilation	0-59	25.28	2.5 (0-14)	0-150	44.13	24 (10-44)	< 0.0001

Duration of antibiotic treatment	0-59	24.42	12 (3-19.5)	3-150	45.13	30 (15-45)	< 0.0001

When the risk factors for symptomatic candidiasis were analyzed surgery was the only independent predictor of symptomatic candidiasis, based on forward stepwise multivariate logistic regression analysis (OR: 6.1; 95% CI: 1.798-20.692). Logistic regression was performed for mortality and risk factors, but a significant association was not noted.

## Discussion

We treated 67 patients with candidiasis in the span of 2 years and 3 months, which constitutes 12% of all PICU admissions during that period-a high percentage, as it comprises both symptomatic and asymptomatic candidiasis cases. In all, 5.9% of PICU admissions were patients with symptomatic candidiasis. Sunit et al. reported that the incidence of candidemia among all PICU admissions was 4.3%, which is similar to the present study's results [5]. Although the incidence of candidiasis in the past has been lower in pediatric departments than in adult departments [6], it has increased during the last decade [7].

Candida albicans was the most common Candida spp. in our patients. When the patients were divided into symptomatic and asymptomatic groups, C. albicans remained the most common among the asymptomatic patients. Although the difference was not significant, the most common Candida spp. in symptomatic patients was Candida parapsilosis. C. albicans is the most common pathogen reported in most studies [5-7]; however, in recent years the proportion of cases due to species other than C. albicans has increased markedly [8]. Similar to the present results, MacDonald et al. reported that 58% of candidemia cases in children were caused by non-albicans Candida spp [9].

C. parapsilosis has emerged as the predominant non-albicans Candida sp. causing candidemia in children [10]. In the present study 48.4% of the symptomatic candidiasis group had C. parapsilosis. It was reported that horizontal transmission of C. parapsilosis from patient to patient, and from the hands of healthcare workers to patients might contribute the high rate of C. parapsilosis isolation [11]. The shift in Candida spp. isolation has also been attributed to the increased use of fluconazole and resistance of non-albicans Candida spp. to fluconazole [12]. The present study's results support this hypothesis, as the most frequent antifungal agent administered to our patients was fluconazole.

A number of risk factors for symptomatic candidiasis in pediatric patients have been reported [13,14] and they were observed in our patients as well. Urinary catheterization and broad-spectrum antibiotic use were the most common risk factors. Weese-Mayer et al. reported that in a neonatal intensive care unit (NICU) tracheal intubation, central catheterization, and total parenteral nutrition were the most common risk factors [15]. On the other hand, Harvey and Myers observed that the most common risk factors in adult patients were central catheterization and blood transfusion [16].

When our symptomatic and asymptomatic patients were compared for these risk factors parenteral nutrition, central venous catheters, and surgery rates were significantly higher in the symptomatic group. Although these factors are common among all patients admitted to a PICU, this emphasizes the need for a high level of suspicion of symptomatic candidiasis in patients with central venous catheters, parenteral nutrition, or surgery. In the present study the risk factors were compared between the symptomatic and asymptomatic groups, and the only risk factor associated with symptomatic candidiasis was surgery. Singhi et al. reported that the presence of colonization and PRISM score were independent predictors of candidemia [17]. This compares well with the present results, as a high rate of colonization has been reported in critically ill surgical patients [18].

Symptomatic candidiasis is associated with a high mortality rate. The overall mortality rate in the present study among the patients with symptomatic candidiasis was 48%. Costa et al. observed an overall mortality rate of 41% in patients with candidemia and 71% in patients with Candida tropicalis infection [19]. Wey at al. observed an excess mortality rate attributable to candidemia of 38%, apart from the underlying disease [20]. Delayed diagnosis due to a lack of pathognomonic symptoms and the absence of reliable rapid diagnostic testing contributes to high mortality. Singhi et al. reported that isolation of non-albicans Candida spp. was significantly associated with mortality; however, in the present study no factor was associated with mortality [5].

It is well known that an ICU admission, especially when prolonged, increases the likelihood of fungal infection [21]. Stamos and Rowley reported that Candida colonization was observed after ≥7 days of hospitalization, which is similar to the present results [6]; however, MacDonald et al. observed Candida colonization in children after a median stay of 25 days [9]. The median length of stay in the PICU before the development of candidemia was 16 days in Singhi et al.'s study, versus 23 days in the present study [17]. Both the duration of PICU stay before Candida growth and total PICU stay were significantly longer in our symptomatic candidiasis group than asymptomatic group; as such, we think the risk of candidiasis increases as time in the PICU increases.

## Conclusions

C. albicans was the most frequent Candida spp. in the present study, but non-albicans Candida spp. were more common among the patients with symptomatic candidiasis. Although surgery was the only risk factor significantly associated with symptomatic candidiasis, other risk factors were commonly observed, and as the length of stay in the PICU increased the risk of candidiasis increased. As symptomatic candidiasis is associated with mortality in the PICU, surveillance blood cultures in high-risk patients and early empiric antifungal therapy may be helpful. Additionally, along with empiric antifungal treatment of symptomatic candidiasis, an increase in the number of non-albicans Candida spp., especially C. parapsilosis, should be considered.

## Competing interests

The authors declare that they have no competing interests.

## Authors' contributions

AEA conceived and drafted the manuscript, NK and BSY have made substantial contributions to conception and design of data. SE performed the statistical analyzes. All authors revised the manuscript for important intellectual content. All authors read and approved the final manuscript.
